# Concurrent strongyloidiasis and allergic bronchopulmonary aspergillosis complicating diagnosis: case report

**DOI:** 10.3389/fmed.2025.1591707

**Published:** 2025-07-24

**Authors:** Zhuangheng Lin, Weiming Su, Dinghui Wu, Yangkun Lin, Lijuan Jian, Zigang He, Dandan Jiang

**Affiliations:** Department of Pulmonary Medicine, Xinglin Hospital of Xiamen, Xiamen, China

**Keywords:** *Strongyloides stercoralis* (*S. stercoralis*), allergic bronchopulmonary aspergillosis (ABPA), elevated IgE, parasitic infections, case report

## Abstract

Herein, we report a case of allergic bronchopulmonary aspergillosis (ABPA) complicated by *Strongyloides stercoralis* infection. A 73-year-old man with a history of duodenal ulcer perforation and prior Billroth II gastrectomy presented with a 14-year history of recurrent cough and wheezing, recently accompanied by new gastrointestinal symptoms. He was initially diagnosed with chronic obstructive pulmonary disease (COPD). Subsequent laboratory evaluation revealed a markedly elevated total serum IgE (1,219 kUA/L) and increased *Aspergillus fumigatus*-specific IgE (0.38 kUA/L, positive cutoff >0.35 kUA/L), leading to a diagnosis of ABPA. Despite antifungal and corticosteroid therapy, total IgE levels remained persistently elevated, accompanied by worsening gastrointestinal symptoms. Multiple stool examinations failed to detect parasitic infection. As gastrointestinal symptoms progressed, gastroscopy revealed the presence of parasitic eggs and larvae. Subsequently, a stool specimen was sent to the Xiamen Center for Disease Control and Prevention. Using the formol-ether concentration technique, the microscopic examination ultimately confirmed an infection with *Strongyloides stercoralis*. Following albendazole treatment, the patient’s symptoms completely resolved. In conclusion, co-infection of ABPA with *Strongyloides stercoralis* may lead to missed or delayed diagnosis due to persistently elevated IgE levels.

## 1 Introduction

*Strongyloides stercoralis* is a soil-transmitted nematode predominantly endemic to tropical and subtropical regions with poor sanitary conditions, affecting an estimated 600 million individuals globally—primarily within rural populations (1). Most infections are asymptomatic or present with only mild symptoms; however, in immunocompromised hosts, the disease can progress to severe forms such as hyperinfection or disseminated strongyloidiasis, both associated with high mortality rates (2–4). Diagnosis of this helminth relies on clinical suspicion, stool examination, and serologic testing; nevertheless, the diagnostic yield is often limited by the variable clinical presentations and the intermittent nature of larval shedding, leading to a substantial risk of missed diagnoses (5–7). Allergic bronchopulmonary aspergillosis (ABPA) is a complex hypersensitivity disorder resulting from immune responses to *Aspergillus fumigatus* (8), and shares numerous clinical features with strongyloidiasis, including respiratory symptoms, peripheral eosinophilia, and elevated IgE levels beyond the normal range (9, 10). When these two conditions coexist, their overlapping manifestations can confound the clinical picture-especially when symptoms are erroneously attributed solely to ABPA-rendering conventional diagnostics inadequate for timely detection of underlying parasitic infection.

Herein, we report a rare case of an elderly male with concomitant *Strongyloides stercoralis* infection and ABPA. The patient was initially diagnosed with ABPA based on characteristic respiratory symptoms and laboratory findings, with no peripheral eosinophilia, thereby obscuring the presence of parasitic infection. It was not until the onset of gastrointestinal symptoms and subsequent gastroscopy, in conjunction with repeated stool microscopy, that *Strongyloides stercoralis* was definitively identified. This case underscores the necessity of heightened clinical vigilance and comprehensive assessment for unexplained, persistent gastrointestinal symptoms, particularly in endemic regions, to prevent misdiagnosis or delayed diagnosis and to ensure prompt, appropriate intervention.

## 2 Case presentation

On October 1, 2024, a 73-year-old male patient presented to the hospital with a 14-year history of recurrent cough, sputum production, and wheezing, accompanied by new-onset abdominal distension and reduced appetite lasting 2 days. He was a long-term resident of a rural area in Fujian Province, an endemic region for *Strongyloides stercoralis* (11). His medical history was notable for duodenal ulcer perforation requiring Billroth II gastrectomy and decades of agricultural work, including frequent barefoot contact with soil. He denied any history of infectious diseases or raw food consumption. Since 2016, he experienced repeated episodes of cough and wheezing, necessitating multiple medical consultations. In February 2024, chest computed tomography (CT) at Xiamen Haicang Hospital revealed severe pulmonary emphysema, multiple bullae, and nodules, leading to a diagnosis of acute exacerbation of chronic obstructive pulmonary disease (COPD). Symptoms temporarily improved following anti-infective and supportive therapies.

On 31 July 2024, the patient experienced another acute exacerbation. Repeat chest CT demonstrated new patchy and band-like opacities in the right upper lobe, with laboratory testing revealing a markedly elevated total IgE level (1,219 kUA/L) and a positive *Aspergillus fumigatus*-specific IgE (ImmunoCAP, Thermo Fisher Scientific) of 0.38 kUA/L (cutoff ≥0.35 kUA/L). He was diagnosed with “COPD complicated by allergic bronchopulmonary aspergillosis (ABPA)” and received voriconazole (200 mg every 12 h) in combination with corticosteroids, resulting in transient improvement of respiratory symptoms. In August, he experienced recurrent episodes of high fever, worsening dyspnea. Chest imaging studies showed multiple linear and nodular opacities in both lungs, along with dilatation of the intrahepatic and extrahepatic biliary ducts.

**Figure 1:**
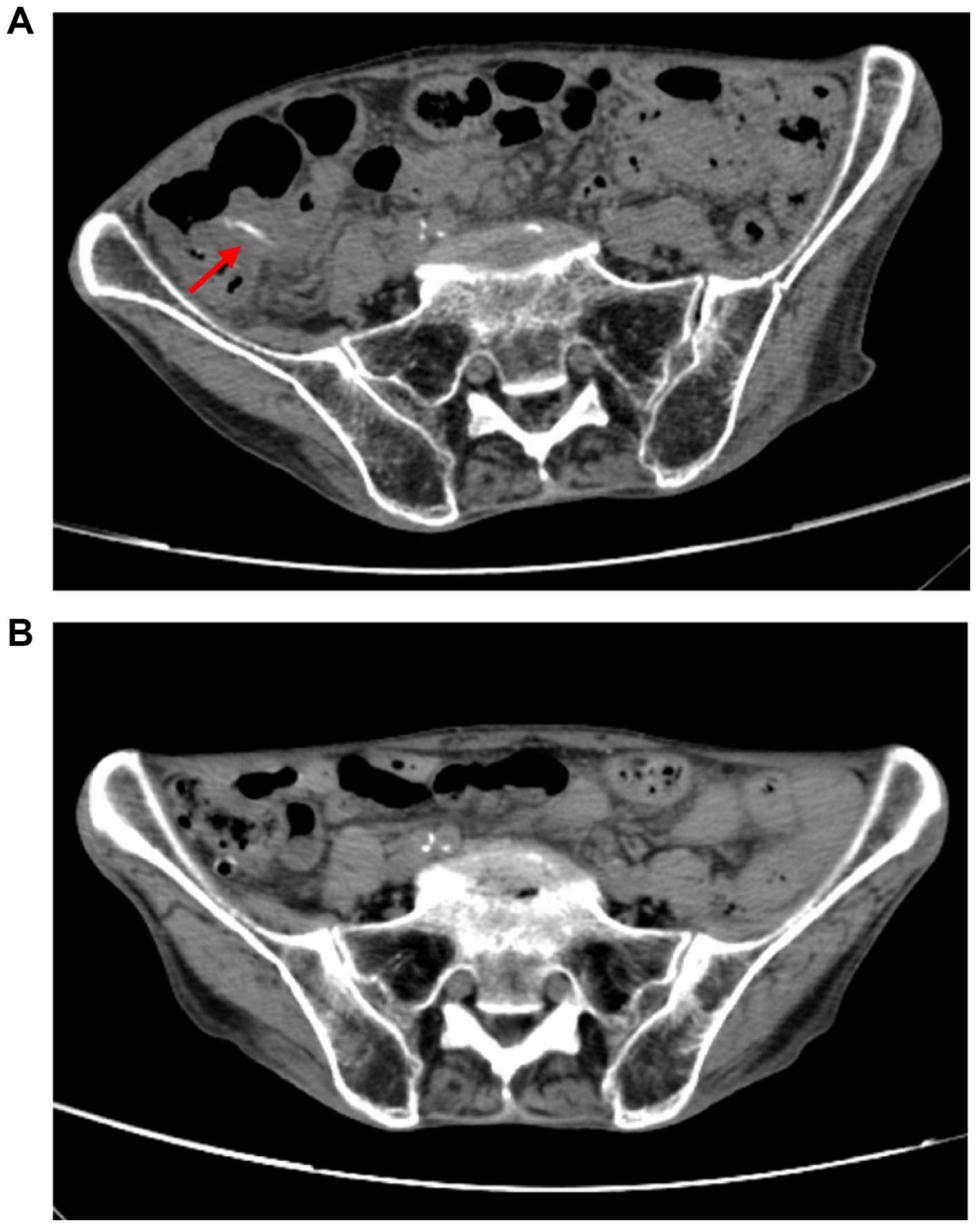
**(A)** A band-like dense shadow exists within the intestinal tract on the right side of the pelvic cavity, suggestive of a possible parasitic infection. **(B)** The strip-like dense shadow within the intestinal tract on the right side of the pelvic cavity has disappeared.

**Figure 2:**
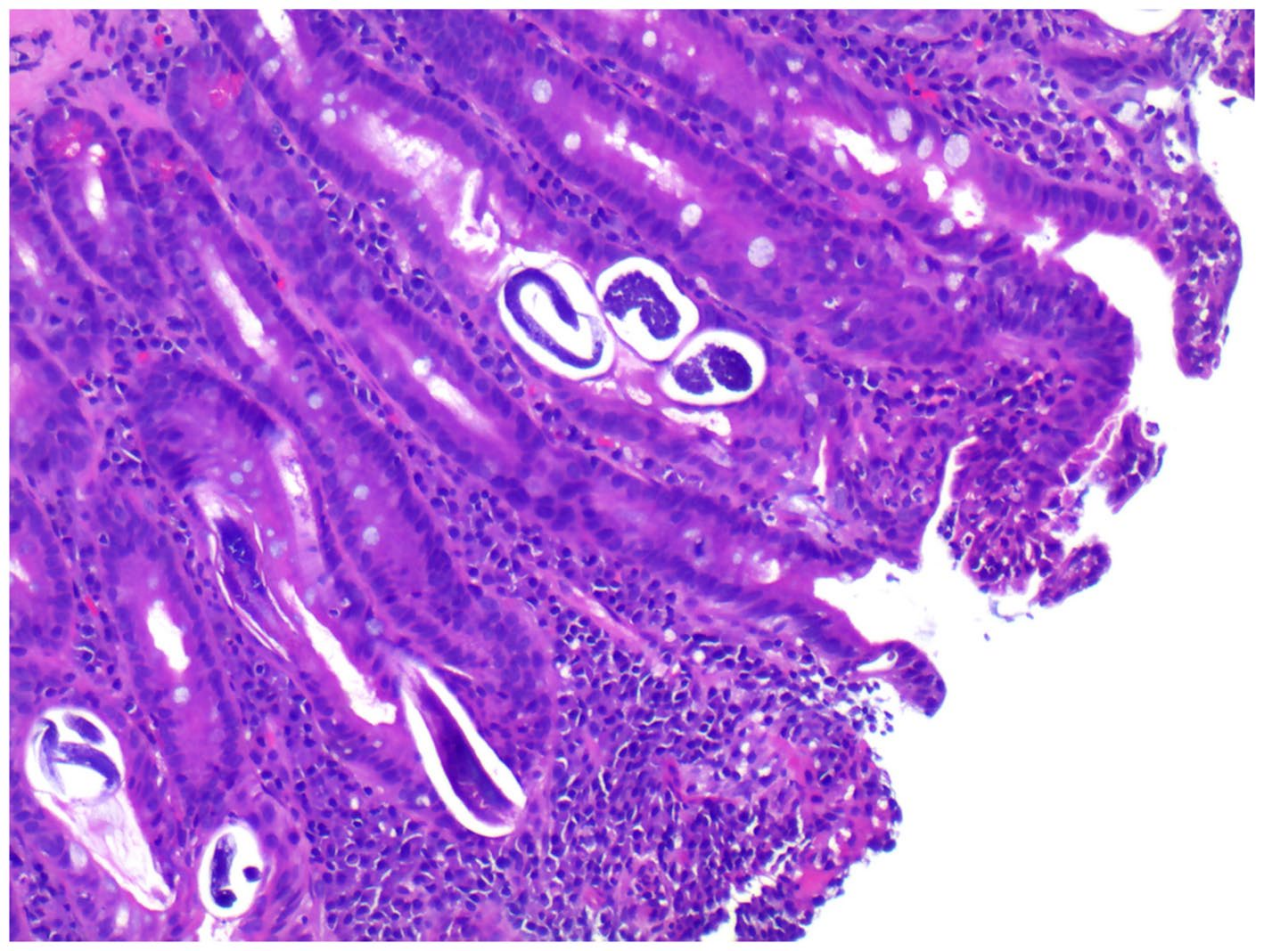
Gastroscopic biopsy reveals thread-like parasites and egg-like structures within the glandular crypts (H&E staining, 40× magnification).

**Figure 3:**
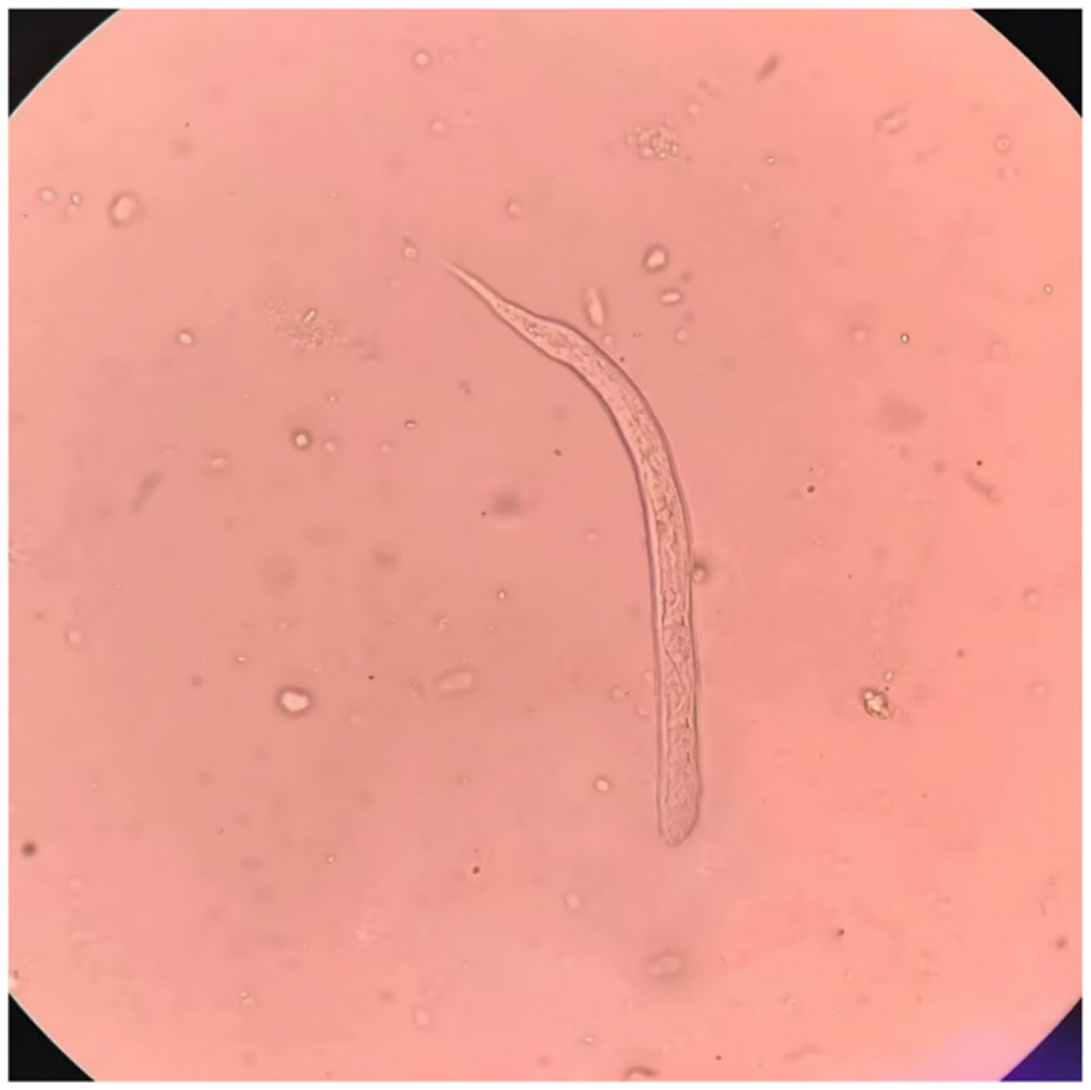
Microscopic examination of feces reveals *Strongyloides stercoralis* (400× magnification).

Upon admission on October 1, 2024, the patient continued to complain of abdominal pain and distension. Supportive care—including fasting, intravenous fluid replacement, and antispasmodic therapy—was initiated. Laboratory parameters on admission are presented in Table 1. Initial stool examinations were unremarkable. Stool examination findings on admission are presented in Table 2. As symptoms persisted and worsened, an abdominal computed tomography (CT) scan was performed, which revealed small gas shadows around the gallbladder (suggestive of possible intraluminal and suspected free air), a small calculus and areas of calcification within the right kidney, prostatic calcification, and a dense linear opacity within the intestinal tract on the right side of the pelvis (Figure 1). Subsequent gastroscopic examination and biopsy revealed the presence of nematodes and ova within the glandular crypts (Figure 2); repeated stool direct microscopy identified live adult worms (Figure 3). Utilizing the formol-ether concentration technique, the Xiamen Center for Disease Control and Prevention ultimately confirmed the diagnosis of *Strongyloides stercoralis* infection.

**Table 1:** Key blood laboratory parameters of the patient.

Parameter	31.07.2024	23.08.2024	01.10.2024	Reference range	Unit
WBC	11.56^a^	12.94^a^	13.02^a^	4–10	×10^9^/L
LYMPH#	0.55^a^	0.75^a^	1.08^a^	1.1–3.2	×10^9^/L
EO#	0.03	0.04	0.05	0.02–0.52	×10^9^/L
BASO#	0.02	0.00	0.00	0–0.06	×10^9^/L
HGB	130	147	145	130–175	g/L
PLT	234	175	244	125–350	×10^9^/L
CD45^+^ Lym#	834.81^a^	749.89^a^	669.45^a^	1,200–3,700	cells/μL
CD3^+^#	341.46^a^	384.51^a^	431.46^a^	520–2,860	cells/μL
CD4/CD8	2.82^a^	2.42^a^	1.71	1.4–2	
CD3^+^ CD4^+^#	243.39^a^	250.26^a^	263.12^a^	400–1,610	cells/μL
CD3^+^ CD8^+^#	86.41^a^	103.45^a^	153.98	150–1,250	cells/μL
CD3–CD19^+^#	184.06	177.70	97.54	70–620	cells/μL
CD3–CD16^+^/CD56^+^#	343.12	180.09	168.96	80–900	cells/μL
PCT	0.184^a^	0.358^a^	0.169^a^	0–0.1	ng/mL
CRP	48.83^a^	9.79^a^	22.79^a^	0–7	mg/L
ALB	36.4^a^	32.9^a^	33.8^a^	40–50	g/L
G test	15.6	10.8	32.9	<100.5	pg/mL
GM test	Neg.	Neg.	Neg.		
M3—specific IgE	0.38^a^	0.07	0.08	<0.35	kUA/L
Total IgE	1219^a^	793^a^	807^a^	0–100	kUA/L
HBsAg	—	—	Pos.		
TP.	—	—	Neg.		
HIV.	—	—	Neg.		
HCV.	—	—	Neg.		

a Abnormal indicators.

—Indicates that testing was not done.

WBC, white blood cell count; NEUT#, neutrophil count; LYMPH#, lymphocyte count; EO#, eosinophil count; BASO#, basophil count; HGB, haemoglobin; PLT, platelets; CD45^+^ Lym#, CD45-positive lymphocyte count; CD3^+^#, CD3-positive cell count; CD3^+^ CD4^+^#, CD3^+^ CD4^+^ T cell count; CD3^+^ CD8^+^#, CD3^+^ CD8^+^ T cell count; CD3–CD19^+^ B lymphocyte count; CD3–CD16^+^/CD56^+^ NK lymphocyte count; PCT, procalcitonin; CRP, c-reactive protein; ALB, albumin; G test, β-D-glucan test; GM test, galactomannan test; HBsAg, hepatitis B surface antigen; TP., *Treponema pallidum* antibody; HIV., Human immunodeficiency virus antibody; HCV., Hepatitis C virus antibody.

GM using ELISA method, *Aspergillus fumigatus* IgE, total IgE using immunofluorescence method.

**Table 2:** Stool examination findings of the patient.

Parameter	31.07.2024	23.08.2024	01.10.2024	16.10.2024	17.10.2024	18.10.2024
Color	Yellow-brown	Yellow-brown	Yellow-brown	Yellow-brown	Yellow-brown	Yellow-brown
Characteristics	Soft	Soft	Soft	Soft	Soft	Soft
Mucus	Neg.	Neg.	Neg.	Neg.	Neg.	Neg.
WBC	0	0	0	0	0	1–3
RBC	0	0	0	0	0	0–2
Fungi	Neg.	Neg.	Neg.	Neg.	Neg.	Neg.
Parasitic eggs	Neg.	Neg.	Neg.	Neg.	Neg.	Pos.
Fat globule	Neg.	Neg.	Neg.	Neg.	Neg.	Neg.
Occult blood test	—	—	—	Neg.	Neg.	Pos.
Stool culture	—	—	—	—	Neg.	—

Following definitive diagnosis, oral albendazole (0.4 g, twice daily for 6 days) and prednisone (10 mg twice daily) were commenced on October 15, 2024. Sequential laboratory assessments revealed that total IgE was 807 kUA/L on October 8, rising to 4,679 kUA/L on October 24. After the combined anti-parasitic and anti-inflammatory regimen, the patient’s abdominal pain and distension markedly improved.

After discharge, the patient was followed up for 6 months. During follow-up, laboratory tests, including total IgE level measurement, were performed every 2 months. The total IgE level gradually decreased to 987 kUA/L on December 1, 2024, and further decreased to 765 kUA/L on February 1, 2025. The patient’s respiratory symptoms remained stable, and no gastrointestinal symptoms recurred. Stool examinations were repeated every 3 months, and no parasites or eggs were detected during the follow-up period.

## 3 Discussion

The risk of strongyloidiasis is significantly increased in patients with immunosuppressive factors, such as hypochlorhydria, leprosy, long-term corticosteroid use, HIV and HTLV-1 infections, anti-tumor necrosis factor α therapy, organ or bone marrow transplantation, and malnutrition (12, 13). Although immunocompetent individuals can also be infected, it is relatively rare. The clinical manifestations of strongyloidiasis are diverse and often nonspecific, such as itching, rash, wheezing, cough, vomiting, abdominal distension, abdominal pain, and constipation. Imaging examinations usually lack specific findings, which poses a significant diagnostic challenge (14). Confirmation requires a comprehensive approach, including stool microscopy, eosinophil counts in blood routine tests, specific antibody ELISA detection, strongyloides antigen ELISA detection, luciferase immunoprecipitation, molecular biological techniques (e.g., PCR, metagenomic second-generation sequencing), gastrointestinal endoscopy biopsy, and histopathological examination (7).

Currently, the main diagnostic methods for strongyloidiasis are stool and histopathological examinations. However, the irregular shedding of parasites in a single stool sample leads to low sensitivity in single stool tests, while multiple stool tests can increase sensitivity to 85%. Additionally, the Baermann egg collection method and agar plate culture method also have high sensitivity (15). Gastroscopy biopsy is of great significance for diagnosis, with a positivity rate of up to 71.4% in duodenal biopsies (14). Thompson et al. (16) reported that taking at least six biopsy samples per lesion resulted in 100% confirmation in six cases.

As of 2024, the co-occurrence of ABPA and strongyloidiasis is extremely rare, with only one case reported (17). This co-occurrence complicates clinical diagnosis, as both can cause elevated IgE levels (9), and strongyloidiasis often presents with atypical symptoms that are easily overlooked. The patient, a rural farmer, had a high risk of infection due to long-term barefoot contact with soil. Chronic malnutrition, a history of duodenectomy, and immunosuppressive therapy for ABPA further weakened his immune defenses, promoting infection.

Although strongyloides is not a common pathogen associated with ABPA, its invasive infection in immunocompromised individuals can accelerate disease progression and significantly increase diagnostic and therapeutic complexity. The clinical manifestations of strongyloidiasis often overlap with ABPA symptoms, leading to misdiagnosis or missed diagnosis. After the use of corticosteroids and immunosuppressants, the infection can rapidly worsen, potentially causing disseminated infection and multi-organ damage. In this case, the patient had multiple immunodeficiency factors, with persistently elevated IgE and specific Aspergillus antibodies. Repeated negative stool tests and atypical gastrointestinal symptoms further complicated clinical recognition and timely treatment.

Standardized diagnosis relies on various detection methods. Repeated stool tests and gastrointestinal endoscopy biopsies are crucial for confirmation. Clinical management should integrate epidemiological exposure history, clinical manifestations, and laboratory findings. Gastroscopy should be performed when necessary to improve detection rates. Comprehensive treatment and follow-up help optimize therapy, reduce complications, and improve prognosis. Although multiple PCR methods show high specificity for strongyloides, most have suboptimal sensitivity (18). The real-time fluorescence PCR method developed by Verweij et al. (19) is widely used but has target specificity issues. A recently reported dual-titer PCR method for strongyloides demonstrates high sensitivity and specificity, with a detection limit of a single larva and no cross-reactivity with other parasites, showing broad application potential (20).

For chronic strongyloidiasis, ivermectin is the first-line treatment at a single oral dose of 200 μg/kg (7). Albendazole and mebendazole are alternatives (21). In this case, albendazole was used and showed effectiveness. This case highlights that in high-prevalence areas, clinicians should remain vigilant to the possibility of chronic strongyloidiasis in patients with multiple immunosuppressive factors, even if stool tests are negative, given the intermittent nature of larval excretion. It underscores the importance of considering the patient’s immunosuppressive status and epidemiological exposure when evaluating such cases.

## 4 Conclusion

ABPA and strongyloidiasis coexistence complicates diagnosis due to overlapping symptoms and elevated IgE. Strongyloidiasis may cause elevated IgE, leading to ABPA misdiagnosis. This case shows the need for clinical vigilance and comprehensive assessment of persistent gastrointestinal symptoms in endemic areas to avoid misdiagnosis. Even with negative stool tests, strongyloidiasis screening is crucial for immunocompromised patients with atypical symptoms due to intermittent larval excretion. Early, accurate diagnosis is essential, and improving parasite detection techniques can prevent misdiagnosis.

## Data Availability

The original contributions presented in the study are included in the article/supplementary material, further inquiries can be directed to the corresponding authors.
